# CoGAPS 3: Bayesian non-negative matrix factorization for single-cell analysis with asynchronous updates and sparse data structures

**DOI:** 10.1186/s12859-020-03796-9

**Published:** 2020-10-14

**Authors:** Thomas D. Sherman, Tiger Gao, Elana J. Fertig

**Affiliations:** 1 Department of Oncology, Johns Hopkins University School of Medicine, Baltimore, MD USA; 2 Department of Computer Science, Johns Hopkins University, Baltimore, MD USA; 3 Department of Applied Mathematics and Statistics, Johns Hopkins University, Baltimore, MD USA; 4 Department of Biomedical Engineering, Johns Hopkins University School of Medicine, Baltimore, MD USA

**Keywords:** Single cell, Matrix factorization, Pattern detection, Unsupervised learning

## Abstract

**Background:**

Bayesian factorization methods, including Coordinated Gene Activity in Pattern Sets (CoGAPS), are emerging as powerful analysis tools for single cell data. However, these methods have greater computational costs than their gradient-based counterparts. These costs are often prohibitive for analysis of large single-cell datasets. Many such methods can be run in parallel which enables this limitation to be overcome by running on more powerful hardware. However, the constraints imposed by the prior distributions in CoGAPS limit the applicability of parallelization methods to enhance computational efficiency for single-cell analysis.

**Results:**

We developed a new software framework for parallel matrix factorization in Version 3 of the CoGAPS R/Bioconductor package to overcome the computational limitations of Bayesian matrix factorization for single cell data analysis. This parallelization framework provides asynchronous updates for sequential updating steps of the algorithm to enhance computational efficiency. These algorithmic advances were coupled with new software architecture and sparse data structures to reduce the memory overhead for single-cell data.

**Conclusions:**

Altogether our new software enhance the efficiency of the CoGAPS Bayesian matrix factorization algorithm so that it can analyze 1000 times more cells, enabling factorization of large single-cell data sets.

## Background

Non-negative matrix factorization (NMF) techniques have emerged as powerful tools to identify the cellular and molecular features that are associated with distinct biological processes from single cell data [3–5, 7, 14, 16]. Bayesian factorization approaches can mitigate local optima and leverage prior distributions to encode biological structure in the features [9, 12]. However, the computational cost of implementing these approaches may be prohibitive for large single cell datasets. Many NMF methods can be run in parallel, and thereby leverage the increasing availability of suitable hardware to scale for analysis of large single cell datasets [1, 8, 10].

Previously, we developed CoGAPS as a sparse, Bayesian NMF approach for bulk [6, 9, 13] and single-cell genomics analysis [3, 11]. Comparison studies to gradient-based NMF [9, 11] and autoencoders [11] demonstrated the unique robustness of this approach to initialization and its inference of dynamic biological processes in bulk and single cell datasets. Further comparison of this approach to principal component analysis and independent component analysis demonstrated the unique ability of this approach to infer transcriptional signatures unique to specific individuals and tissues in GTEx [12]. CoGAPS was designed to perform Gibbs sampling for a unique prior distribution that adapts the level of sparsity to the distribution of expression values in each gene and cell. While this design allows CoGAPS to adapt to different types of data, it also imposes a constraint on the algorithm that requires the update steps to be proposed sequentially. While the sequential updates of CoGAPS limit implementation of embarrassingly parallel computational approaches, we present a new method for isolating the sequential portion of CoGAPS so that the majority of the algorithm can be run in parallel. Additionally, we derive an optimization for sparse data in order to take advantage of the nature of many single-cell data sets. In combination, these new features in CoGAPS version 3.2 allows for efficient Bayesian NMF analysis of large single cell data sets.

## Implementation

### The CoGAPS algorithm

The input for CoGAPS is a data matrix of single-cell data with N measures (e.g., genes, genomic coordinates, proteins) and M cells, $D \in {\mathbb{R}}^{N \times M}$, and a number of patterns (features) to learn, $K$. It factors $D$ into two lower dimensional matrices, $A \in {\mathbb{R}}^{N \times K}$ and $P \in {\mathbb{R}}^{K \times M}$. The columns of the $A$ matrix contains relative weights of each measurement for the learned features and the corresponding rows of the $P$ matrix contains the relative expression of those features in each cell [12]. CoGAPS assumes the elements of $D$ are normally distributed with mean $AP$ and variance proportional to $D$. The algorithm has a Gamma prior on each element of $A$ and $P$ whose shape hyper parameter has a Poisson prior. This model encodes a sparsity constraint that adapts to the relative sparsity of each gene or cell in the data [11]. In version 3.2 of CoGAPS, the input matrix $D$ can now be passed as either a data matrix or a Single Cell Experiment object. The output for $A$ and $P$ is stored in a Linear Embedding Matrix to enable compatibility with emerging single-cell workflows Bioconductor.

### Asynchronous updates

Although the algorithm that determines the order of the matrix updates in CoGAPS must be run sequentially, the large number of measurements in genomics data provide feasibility for running the most computationally intensive portion of the algorithm in parallel. Notably, the proposal for which matrix element to update can be made efficiently, whereas evaluating the new value for that element requires an expensive calculation across a row or column of the data. We take advantage of this fact with an asynchronous updating scheme that yields a Markov chain that is equivalent to the one obtained from the standard sequential algorithm [6]. In order to do this, we build up a queue of proposals using the sequential algorithm until a conflicting proposal is generated, at which point we evaluate the entire queue of proposals in parallel.

**Fig. 1:**
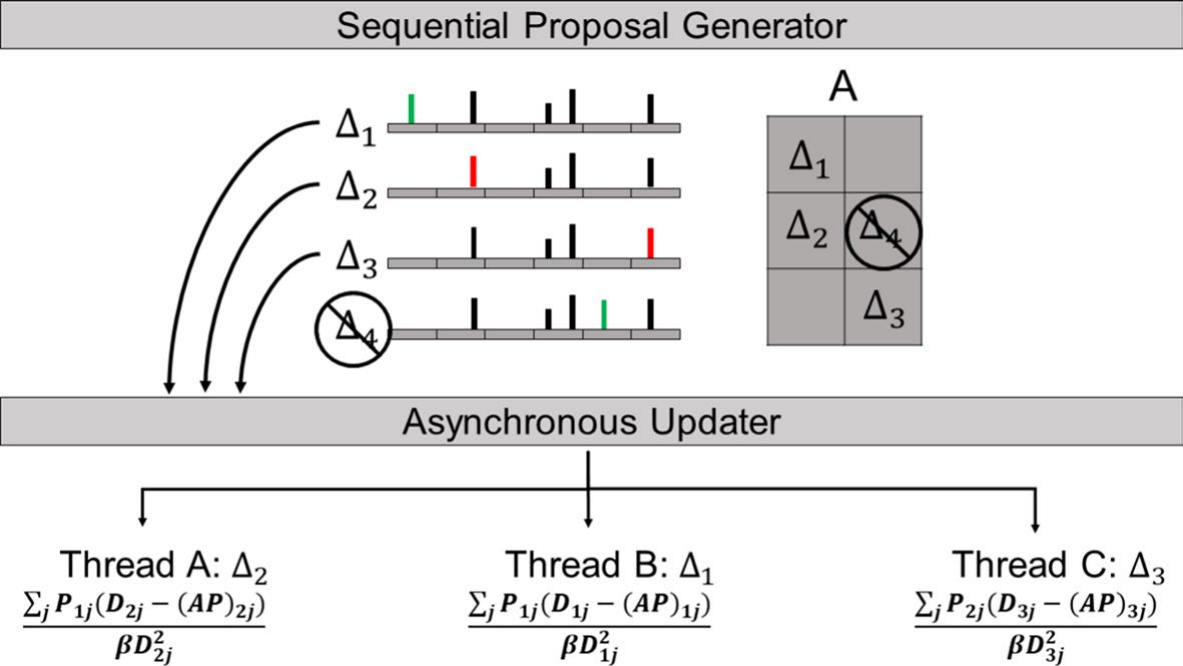
Schematic for the asynchronous updating scheme used in CoGAPS. Updates are proposed sequentially until a conflicting proposal is generated, at which time the existing queue of proposals is evaluated in parallel. In this case, proposed change #4 is conflicting since it is in the same row as #2. When evaluating a proposal, an entire row or column of $AP$ is used in the calculation of the conditional distribution used to perform Gibbs sampling. When a change is made in row $n$ of $A$, the entire *n*th row of $AP$ changes. So, if another change is later proposed in row $n$, the value of $AP$ used will depend on the previous proposal thereby changing the conditional distribution for this new proposal. This is exactly the case here for #2 and #4. Changes #1, #2, #3 can be processed in parallel since they do not conflict with each other

The asynchronous updating scheme heavily relies on the designation of conflicting, or dependent, proposals. Specifically, if two proposals are independent, they must be able to be evaluated in any order without one impacting the sampling distribution of the other. This allows a queue of independent proposals to be evaluated in parallel and still produce a deterministic result. One example of dependent proposals is given here (Fig. 1) and a full accounting of all possible conflicts can be found in Additional file 1.

### Sparse data structures

Single-cell data tends to be sparse. Therefore, the natural solution for reducing memory overhead is to use sparse data structures to represent the data $D$ in the analysis. While the data, $D$, may be very sparse, the weights in $A$ and $P$ correct for dropout and therefore have a product that may be largely non-zero. Traditionally, CoGAPS caches this product to reduce the number of calculations at each step. However, caching $AP$ introduces an unacceptable memory overhead when the data is stored in a sparse format. To address this, we separate the matrix calculations into terms that can be efficiently calculated using only the non-zero entries of $D$ and terms that can be precomputed before each batch of updates as described in detail in Additional file 1. By doing this, we can make the computation time proportional to the sparsity of the data. However, since storing the data in a sparse format requires the calculation of $AP$ during the update steps, there is a performance trade-off that needs to be considered. Typically, when the data is more than 80% sparse it is more efficient to use the sparse optimization, even though it requires calculating $AP$.

## Results

We simulated three sparse single-cell datasets with the R package Splatter [15]. We varied the level of sparsity in each data set and tested the amount of memory used with and without the sparse optimization enabled. We also measured the run time in both the single-threaded and multi-threaded case. Table 1 gives a high-level overview of the performance differences. For example, with 2000 genes and 2000 cells when the data is 90% zeroes, using the sparse optimization and 4 threads will give identical results to the standard algorithm in 1/5 of the time while using 1/25 of the memory.

**Table 1:** Relative performance of the sparse optimization on 2000 genes and 2000 cells, baseline is the standard algorithm with 1 thread and no sparse optimization

Data sparsity (%)	Memory (MB)	Runtime (1 thread)	Runtime (4 threads)
70	0.14	1.92	0.62
80	0.09	1.24	0.42
90	0.04	0.42	0.20

**Fig. 2:**
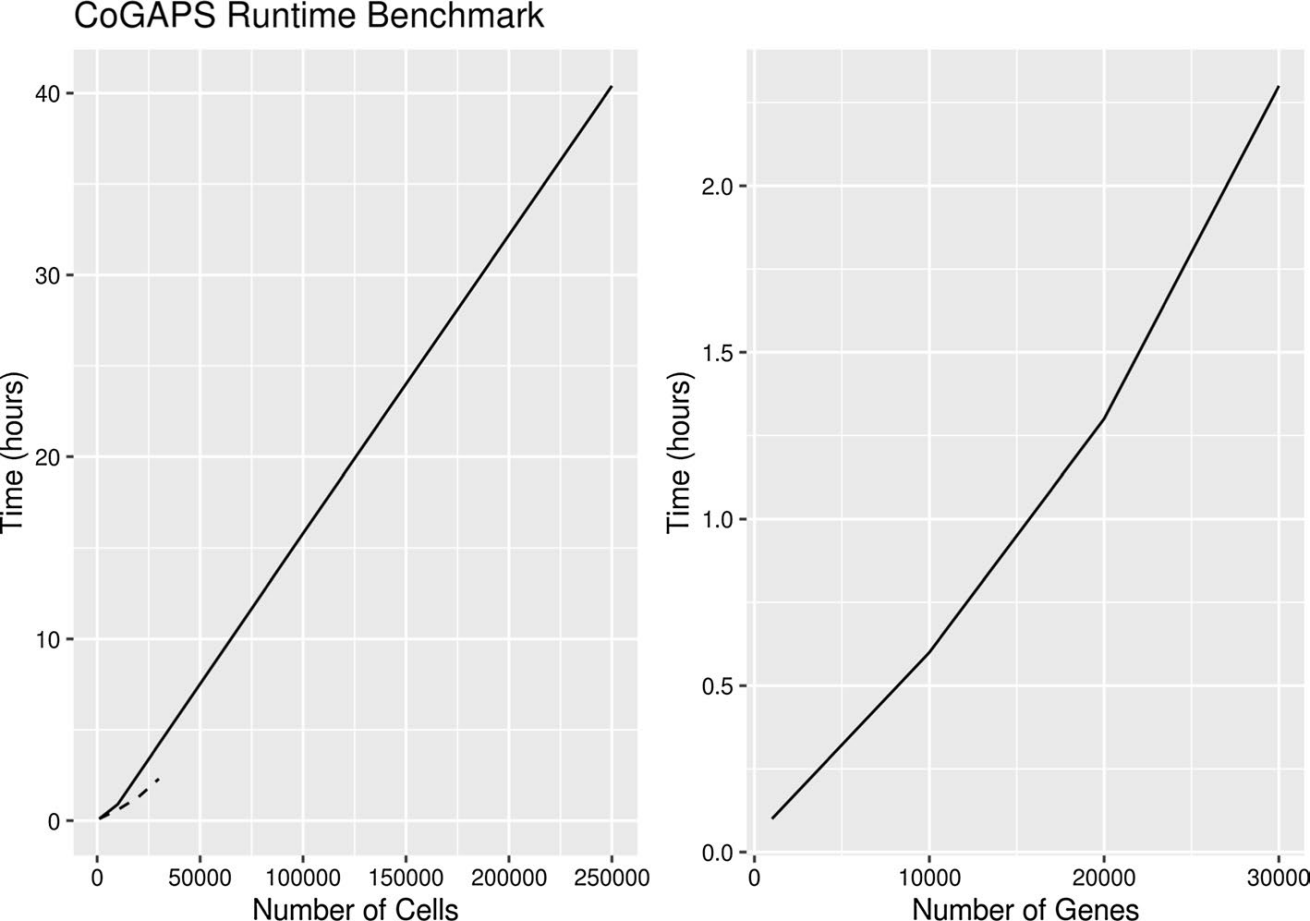
Run time (in hours) of CoGAPS on random subsets of cells for a subset of 1000 genes (left) and random subsets of genes for a subset of 1000 cells (right) from the Li et al. [8] umbilical cord blood single cell dataset. Dotted line in the left plot corresponds to the values in the right plot and is included for scale reference

We also tested the performance on a single-cell 10X data set generated at the Broad Institute [2]. We ran a benchmark on a subset of 37,000 human immune cells (umbilical cord blood) and only kept the 3000 highest variance genes. In this case, enabling the sparse optimization reduced memory overhead by 82% and cut the run time by 74%. When using 4 threads instead of 1, the run time was cut by an additional 36%. We further ran this benchmark on random subsets of this data to benchmark performance as a function of number of genes and number of cells (Fig. 2). We observe that the running time in these simulations increases linearly with both the number of cells and the number of genes as expected.

## Conclusions

In this paper, we present an algorithm and software to enable parallelization of CoGAPS to enable analysis of large single cell datasets. This parallelization was done by combining existing methods for Gibbs sampling [1, 8, 10] with a new infrastructure for the updating steps in CoGAPS. Prior to the implementation of an asynchronous updating scheme, CoGAPS was applied to large data sets by using a distributed version of the algorithm, GWCoGAPS, that performed analysis across random sets of genes [13] or random sets of cells [11]. This distributed version leveraged the observation that the learned values of $A$ and $P$ are robust across these random sets. Future work combining the asynchronous and distributed parallelization methods will be critical to further enhance performance by utilizing all CPU cores efficiently.

## Availability and requirements

- Project name: CoGAPS
- Project home page: https://doi.org/doi:10.18129/B9.bioc.CoGAPS
- Operating systems: Platform independent
- Programming languages: R and C++
- Other requirements: R version 3.6 or higher
- Any restrictions to use by non-academics: None

## Supplementary information

**Additional file 1:** Extended methods describing the asynchronous update algorithm to enhance parallelization and efficiency for the Bayesian NMF in Version 3.0.

## Data Availability

The datasets analyzed during the current study are available from Bo Li et al*.* Census of Immune Cells. Broad Inst. Mass. Inst. Technol. Howard Hughes Med. Inst. Retrieved from https://data.humancellatlas.org/explore/projects/cc95ff89-2e68-4a08-a234-480eca21ce79.

## Abbreviations

CoGAPS: Coordinated Gene Activity in Pattern Sets
CPU: Central processing unit
NMF: Non-negative matrix factorization
